# Photoactivated UVR8-COP1 Module Determines Photomorphogenic UV-B Signaling Output in *Arabidopsis*

**DOI:** 10.1371/journal.pgen.1004218

**Published:** 2014-03-20

**Authors:** Xi Huang, Panyu Yang, Xinhao Ouyang, Liangbi Chen, Xing Wang Deng

**Affiliations:** 1 Peking-Yale Joint Center for Plant Molecular Genetics and Agro-Biotechnology, State Key Laboratory of Protein and Plant Gene Research, Peking-Tsinghua Center for Life Sciences, College of Life Sciences, Peking University, Beijing, China; 2 Department of Botany, College of Life Sciences, Hunan Normal University, Changsha, China; 3 Department of Molecular, Cellular, and Developmental Biology, Yale University, New Haven, Connecticut, United States of America; The University of North Carolina at Chapel Hill, United States of America

## Abstract

In *Arabidopsis*, ultraviolet (UV)-B-induced photomorphogenesis is initiated by a unique photoreceptor UV RESISTANCE LOCUS 8 (UVR8) which utilizes its tryptophan residues as internal chromophore to sense UV-B. As a result of UV-B light perception, the UVR8 homodimer shaped by its arginine residues undergoes a conformational switch of monomerization. Then UVR8 associates with the CONSTITUTIVELY PHOTOMORPHOGENIC 1-SUPPRESSOR OF PHYA (COP1-SPA) core complex(es) that is released from the CULLIN 4-DAMAGED DNA BINDING PROTEIN 1 (CUL4-DDB1) E3 apparatus. This association, in turn, causes COP1 to convert from a repressor to a promoter of photomorphogenesis. It is not fully understood, however, regarding the biological significance of light-absorbing and dimer-stabilizing residues for UVR8 activity in photomorphogenic UV-B signaling. Here, we take advantage of transgenic UVR8 variants to demonstrate that two light-absorbing tryptophans, W233 and W285, and two dimer-stabilizing arginines, R286 and R338, play pivotal roles in UV-B-induced photomorphogenesis. Mutation of each residue results in alterations in UV-B light perception, UVR8 monomerization and UVR8-COP1 association in response to photomorphogenic UV-B. We also identify and functionally characterize two constitutively active UVR8 variants, UVR8^W285A^ and UVR8^R338A^, whose photobiological activities are enhanced by the repression of CUL4, a negative regulator in this pathway. Based on our molecular and biochemical evidence, we propose that the UVR8-COP1 affinity in plants critically determines the photomorphogenic UV-B signal transduction coupling with UVR8-mediated UV-B light perception.

## Author Summary

Higher plants are able to sense and interpret diverse light signals to modulate their growth. In response to long-wavelength and low-intensity ultraviolet-B (UV-B) light, plants establish photomorphogenic development and stress acclimation. UV RESISTANCE LOCUS 8 (UVR8) is a unique UV-B photoreceptor that triggers photomorphogenesis in *Arabidopsis thaliana*. However, the signaling process following UV-B light perception by plants is not fully understood. In this study, by generating transgenic UVR8 variants in *Arabidopsis*, we have extensively analyzed the biological significance of key residues in UVR8 for UV-B-induced photomorphogenesis. Furthermore, by engineering and characterizing two constitutively active UVR8 variants, we have provided the biochemical insight that the *in vivo* association between UVR8 and CONSTITUTIVELY PHOTOMORPHOGENIC 1 (COP1) critically determines the photomorphogenic UV-B signaling output.

## Introduction

Light is a critical environmental stimulus that regulates a number of developmental and physiological processes of living organisms. In sessile plants, perception of light is the initial and decisive step in light signaling transduction, and is achieved by several groups of photosensory receptor proteins. Phytochromes sense far-red and red light [1], [2]. Cryptochromes and phototropins perceive blue and ultraviolet (UV)-A light [3], [4], [5], [6]. In *Arabidopsis thaliana*, UV RESISTANCE LOCUS 8 (UVR8) has recently been identified as a photoreceptor that detects UV-B (280 to 320 nm) light [7]. Long-wavelength and low-fluence UV-B induces plant photomorphogenic development that is physically characterized by the inhibition of hypocotyl elongation, flavonoid accumulation, and UV-B stress tolerance [8], [9], [10], [11].

*UVR8* was originally isolated as a UV-resistance gene, having been shown to contribute to the UV-B-induced flavonoid accumulation and UV-B protection [12]. Transcriptomic analyses have revealed that UVR8 positively orchestrates UV-B signaling specifically under photomorphogenic UV-B [13]. Later, a series of functional studies have disclosed that UVR8 exhibits a number of features characteristic of photoreceptors, including a broad-range loss of UV-B responsive gene expression in the *uvr8* null mutant [13], [14], the enrichment of aromatic residues in UVR8 protein and UV-B-induced conformational change of UVR8 [7].

Despite these insights, however, the exact process by which UVR8 mediates UV-B light perception remained unclear until two research groups independently reported the structure of UVR8 [15], [16]. Without the presence of UV-B light, UVR8 appears as a symmetric seven-bladed-β-propeller homodimer that is stabilized by arginines primarily Arg 286 and Arg 338. These arginine residues shape intramolecular cation-π interactions with their surrounding tryptophan residues, among which Trp 285 and Trp 233 act as the internal UV-B chromophore. Consequently, unlike phytochromes and cryptochromes, UVR8 is devoid of external small molecules as chromophore. Upon UV-B irradiation, the dark-state dimer of UVR8 is monomerized as a result of the disruption of the intramolecular cation-π interactions and the intermolecular hydrogen bonds mediated by Arg 286 and Arg 338 [7], [15], [16]. This structural conversion, which takes place in seconds, is a major determinant for UVR8 to sequester CONSTITUTIVELY PHOTOMORPHOGENIC 1-SUPPRESSOR OF PHYA (COP1-SPA) core complex(es) from the CULLIN 4-DAMAGED DNA BINDING PROTEIN 1 (CUL4-DDB1) E3 apparatus. Ultimately, this complex reorganization enables COP1 to act as a positive regulator in the UV-B-induced photomorphogenesis by facilitating the stability and activity of a photomorphogenesis-promoting transcription factor ELONGATED HYPOCOTYL 5 (HY5) [7], [17]. Reversibly, upon the elimination of UV-B irradiation, REPRESSOR OF UV-B PHOTOMORPHOGENESIS 1 (RUP1) and RUP2, two UVR8-interacting proteins, might disrupt the physical contact of UVR8 and COP1, so that UVR8 dimerization can be regenerated [7], [15], [16], [18], [19], [20]. However, the exact biological significance of key residues in UVR8 has not been fully determined to date.

Here we take advantage of site-directed mutagenesis to generate UVR8 variant proteins in *Arabidopsis*, and demonstrate the pivotal roles of two light-absorbing tryptophans, W233 and W285, and two dimer-stabilizing arginines, R286 and R338 in UVR8-initiated UV-B-induced photomorphogenesis. We also characterize two constitutively active forms of UVR8, UVR8^W285A^ and UVR8^R338A^, whose photobiological activity is enhanced when the repressor CUL4 is suppressed. Overall, our molecular and biochemical evidence has supported that the intrinsic affinity of UVR8-COP1 critically determines the efficiency of photomorphogenic UV-B signal transduction coupling with UVR8-mediated UV-B light perception.

## Results

### UVR8 variants differentially interact with COP1 in yeast

**Figure 1:**
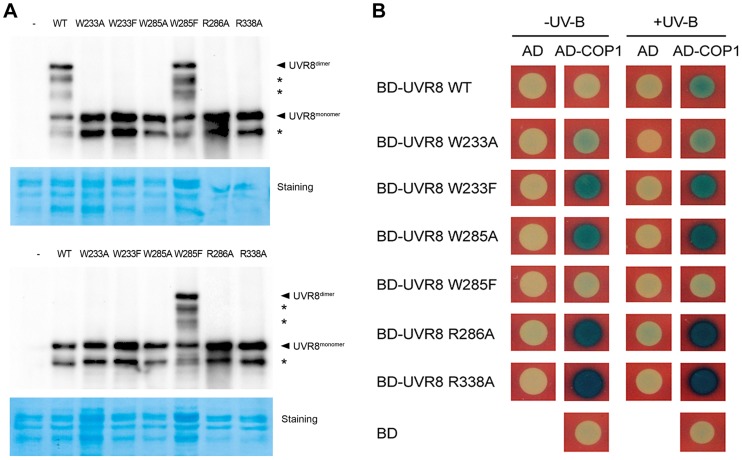
UVR8 variants display altered interaction with COP1 in yeast. (A) Conformational status of wild-type and mutated UVR8 proteins in yeast. Total proteins of yeast expressing LexA fused wild-type and mutated UVR8 were extracted and incubated under −UV-B and +UV-B for 20 min. Protein samples without heat denaturation were assayed in SDS-PAGE and immunoblot analysis by anti-LexA antibody. Staining by Coomassie brilliant blue (CBB) is shown as a loading control. The asterisks indicate unspecific degradation products. (B) Interaction of wild-type and mutated UVR8 proteins with COP1 in yeast two-hybrid assays. Transformants in the respective combinations were incubated under −UV-B and +UV-B for 16 h.

In order to assess the roles of key residues in UVR8, we generated six UVR8 variants in two groups based on their functional classification via site-directed mutagenesis. The first group, which included UVR8^W233A^, UVR8^W233F^, UVR8^W285A^ and UVR8^W285F^, and the second group, which included UVR8^R286A^ and UVR8^R338A^, were designed to interrupt UVR8's perception of UV-B light and dimer stabilization respectively (Figure S1A). As yeast has been widely used as an efficient system to determine the conformational status of UVR8 and its interaction with other proteins in response to UV-B [7], [21], [22], we first introduced wild-type and these six mutated UVR8 (mUVR8) into yeast. Using SDS-polyacrylamide gel electrophoresis (SDS-PAGE) followed by immunoblot analyses, we found that UVR8^WT^ was dimeric under −UV-B and monomeric under +UV-B (Figure 1A), while UVR8^W233F^, UVR8^W285A^ and UVR8^W285F^ were constitutively monomeric, monomeric and dimeric respectively (Figure 1A). This is consistent with the results reported previously [7]. The other UVR8 variants, UVR8^W233A^, UVR8^R286A^ and UVR8^W338A^ appeared as monomers irrespective of UV-B treatment (Figure 1A). These results demonstrate that in yeast, in addition to the dimer-stabilizing arginines, R286 and R338, the light-absorbing tryptophans, W233 and W285, critically contribute to the dimer-to-monomer switch of UVR8 upon UV-B irradiation.

We next performed a series of yeast two-hybrid assays in order to examine the effects of the UVR8 mutations on the interaction between UVR8 and COP1. While COP1 only interacted with UVR8^WT^ under +UV-B, it interacted with UVR8^W233A^, UVR8^W233F^, UVR8^W285A^, UVR8^R286A^ and UVR8^R338A^ under both −UV-B and +UV-B. The interaction between UVR8^W285F^ and COP1 was barely observed (Figure 1B). These results suggest that the UVR8-COP1 interaction in yeast requires UVR8 to be in its monomeric form. Furthermore, previous studies have also proposed that RUP1 and RUP2 interact with UVR8 independent of UV-B irradiation [19]. Specifically, it has been suggested that these two proteins mediate UVR8 redimerization and disrupt UVR8-COP1 interaction, so as to facilitate the inactivation of the photoreceptor [18]. In yeast, RUP1 was found to constitutively interact with UVR8^W233A^, UVR8^W233F^, UVR8^W285A^ and UVR8^R286A^, but was not observed to interact with UVR8^WT^, UVR8^W285F^ and UVR8^R338A^. Differently, RUP2 was observed to interact with the wild-type UVR8 and all the mutant UVR8 proteins (Figure S1B). These results collectively suggest that light perception and conformational change of UVR8 are both major determinants of its interaction with key players of UV-B-induced photomorphogenesis.

### UVR8 variants alter physiological responses to photomorphogenic UV-B in *Arabidopsis*

**Figure 2:**
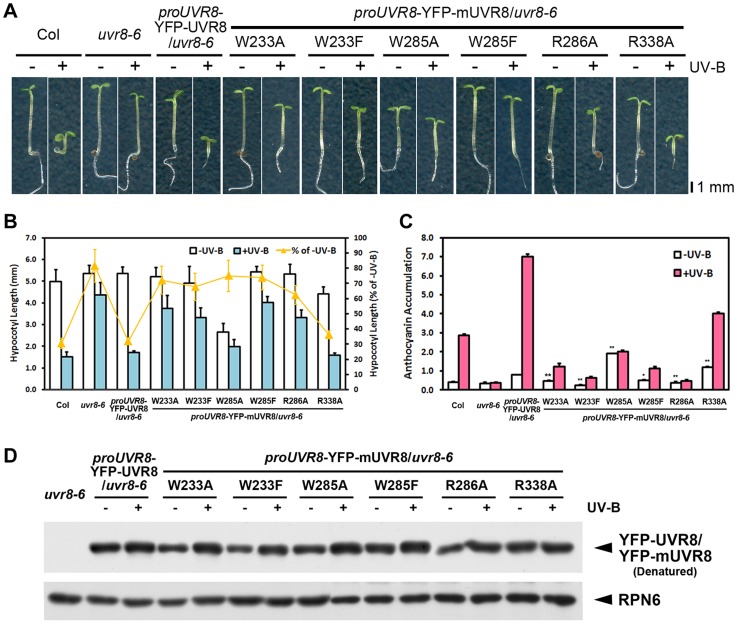
UVR8 variants result in impaired UV-B-induced photomorphogenesis. (A) Phenotypes of 4-day-old seedlings of transgenic UVR8 variant lines grown under −UV-B and +UV-B. (B) Hypocotyl length of the seedlings shown in (A). Data are shown as mean ± SD; n>30. (C) Anthocyanin content of the seedlings shown in (A). Data are shown as mean ± SD; n = 3. The difference significance of UVR8 variants from wild-type UVR8 in anthocyanin content under −UV-B was analyzed by Student's t test. *, p<0.05. **, p<0.01. (D) Immunoblot assay of wild-type and mutated UVR8 proteins (by anti-GFP antibody) in 4-day-old transgenic seedlings grown under −UV-B and +UV-B. Anti-RPN6 was used as a loading control.

To investigate the biological activity of these UVR8 variants in *Arabidopsis*, we introduced wild-type and mutated UVR8 fused with yellow fluorescent protein (YFP) driven by the native *UVR8* promoter into the *uvr8* null mutant *uvr8-6* background. As expected, *proUVR8*-YFP-UVR8/*uvr8-6* (YFP-UVR8^WT^) was found to express the transgenic UVR8 protein at a level comparable to the endogenous UVR8 protein in Col under −UV-B and +UV-B (Figure S2A). It was able to rescue *uvr8-6* in UV-B-induced hypocotyl growth and anthocyanin accumulation (Figures 2A, 2B and 2C) which are characteristic physiological responses to photomorphogenic UV-B [14], [23]. We next examined these two responses in the six transgenic UVR8 variants expressing YFP-UVR8 proteins at equivalent levels to that in YFP-UVR8^WT^ (Figure 2D). The hypocotyl shortening in all the variants failed to reach the shortening degree detected in YFP-UVR8^WT^. Interestingly, YFP-UVR8^W285A^ and YFP-UVR8^R338A^ displayed shorter hypocotyl than YFP-UVR8^WT^ under −UV-B, while YFP-UVR8^R338A^ instead of YFP-UVR8^W285A^ showed further shortened hypocotyl under +UV-B (Figures 2A and 2B). Furthermore, compared with YFP-UVR8^WT^, reduced anthocyanin accumulation was found in YFP-UVR8^W233A^, YFP-UVR8^W233F^, YFP-UVR8^W285F^ and YFP-UVR8^R286A^ under both −UV-B and +UV-B. In contrast, enhanced anthocyanin accumulation was detected in YFP-UVR8^W285A^ and YFP-UVR8^R338A^ under −UV-B. YFP-UVR8^R338A^ was even able to accumulate anthocyanin under +UV-B, though at a level lower than that in YFP-UVR8^WT^ (Figure 2C).

These observations showed that all the mutations interfered with UV-B-induced photomorphogenesis, though to varying degrees. The mutations in UV-B-absorbing residues resulted in more severe phenotypic defects than those in UVR8-dimerizing residues, suggesting the importance of the sequential action of UV-B light perception before UVR8 monomerization. Meanwhile, a specific activity of UVR8^W285A^ and UVR8^R338A^ is indicated by the constitutive short hypocotyl and high anthocyanin content found in YFP-UVR8^W285A^ and YFP-UVR8^R338A^ (Figures 2B and 2C).

### UVR8 mutations affect UV-B-responsive gene expression

**Figure 3:**
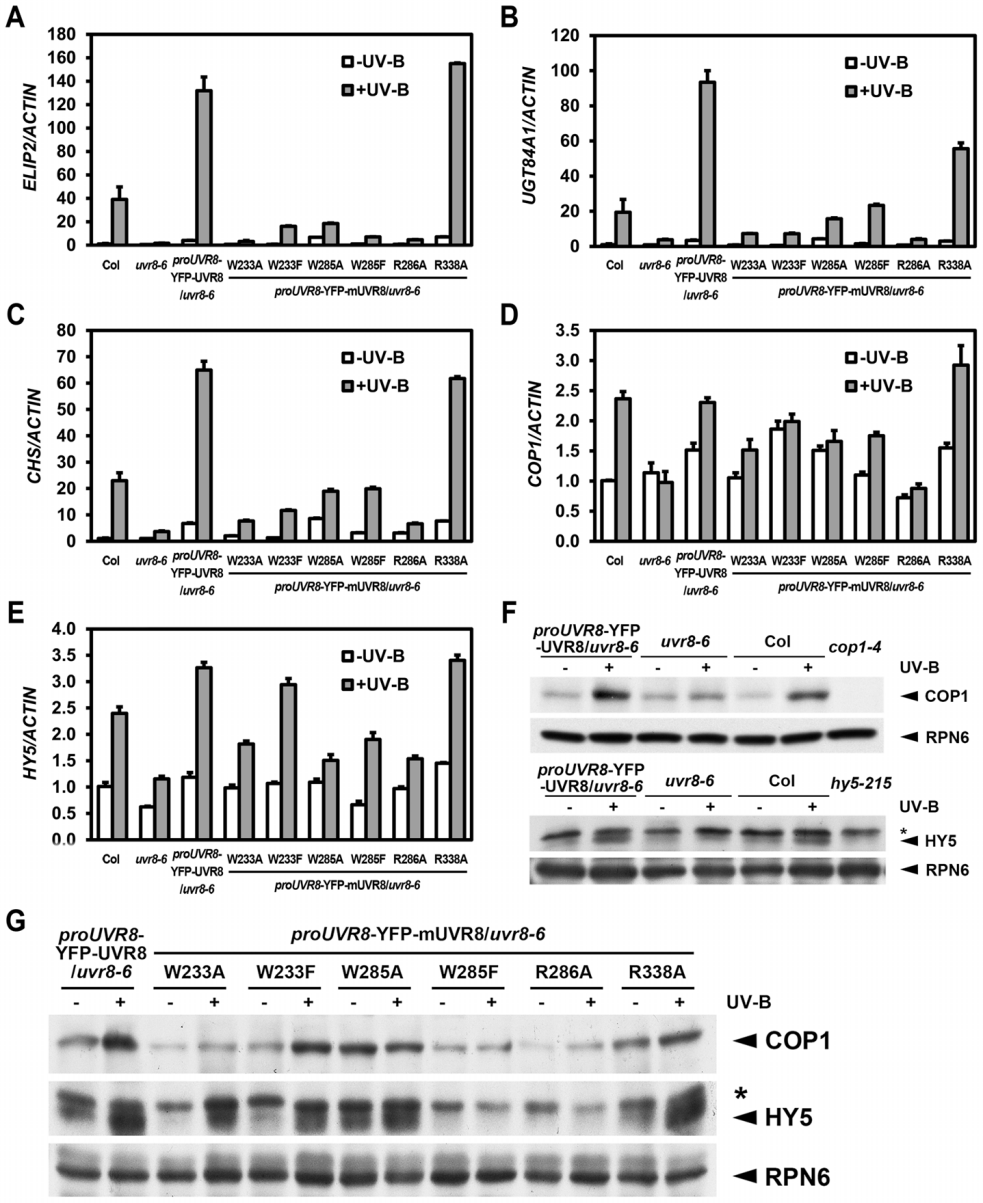
UVR8 mutations lead to abnormal UV-B-responsive gene expression. (A–E) qRT-PCR analysis of UV-B-responsive gene expression *ELIP2* (A), *UGT84A1* (B), *CHS* (C), *COP1* (D) and *HY5* (E) in 4-day-old seedlings grown under −UV-B and +UV-B. Data are shown as mean ± SD; n = 3. (F–G) Immunoblot assay of COP1 and HY5 proteins (by anti-COP1 and anti-HY5 antibodies) in 4-day-old seedlings grown under −UV-B and +UV-B. Anti-RPN6 was used as a loading control. The asterisk indicates an unspecific cross-reactive band.

It is well known that differential transcriptomic regulation is orchestrated by photomorphogenic UV-B in *Arabidopsis* [9], [13]. Using 4-day-old seedlings grown under −UV-B and +UV-B, we examined the expression pattern of several UV-B-responsive marker genes, *EARLY LIGHT-INDUCIBLE PROTEIN 2* (*ELIP2*), *UDP-GLYCOSYLTRANSFERASE 84A1* (*UGT84A1*) and *CHALCONE SYNTHASE* (*CHS*). The UV-B-induced activation of these genes observed in YFP-UVR8^WT^ was largely impaired in YFP-UVR8^W233A^, YFP-UVR8^W233F^, YFP-UVR8^W285A^, YFP-UVR8^W285F^ and YFP-UVR8^R286A^, while the activation was readily detected in YFP-UVR8^R338A^ though it showed a reduced induction of *UGT84A1*. Again, we noticed YFP-UVR8^W285A^ and YFP-UVR8^R338A^ as these two variants accumulated higher transcript levels of these genes than YFP-UVR8^WT^ under −UV-B (Figures 3A, 3B and 3C).

In addition to these marker genes, key regulators of UV-B specific signaling, including the positive regulators *COP1* and *ELONGATED HYPOCOTYL 5* (*HY5*) are also transcriptionally governed by photomorphogenic UV-B [24], [25]. We found that the accumulation of *COP1* mRNA was diminished in all UVR8 variants with the exception of YFP-UVR8^R338A^ (Figure 3D). Similarly, though the accumulation of *HY5* mRNA was observed in YFP-UVR8^W233F^ and YFP-UVR8^R338A^, it was limited in YFP-UVR8^W233A^, YFP-UVR8^W285A^, YFP-UVR8^W285F^ and YFP-UVR8^R286A^ (Figure 3E). At the post-transcriptional level, COP1 protein was clearly induced in YFP-UVR8^WT^, YFP-UVR8^W233F^ and YFP-UVR8^R338A^, slightly increased in YFP-UVR8^W233A^ under +UV-B, and relatively high under both −UV-B and +UV-B in YFP-UVR8^W285A^. However, the accumulation of COP1 protein mediated by photomorphogenic UV-B was scarcely detected in YFP-UVR8^W285F^ and YFP-UVR8^R286A^ (Figures 3F and 3G). A similar pattern was observed for the case of HY5 protein accumulation (Figures 3F and 3G). Taken together, the altered expression profiles of these UV-B responsive genes suggest that these residues responsible for UV-B light perception and UVR8 dimerization are crucial for UVR8 activity in the transcriptional control of UV-B signaling at a molecular level.

**Figure 4:**
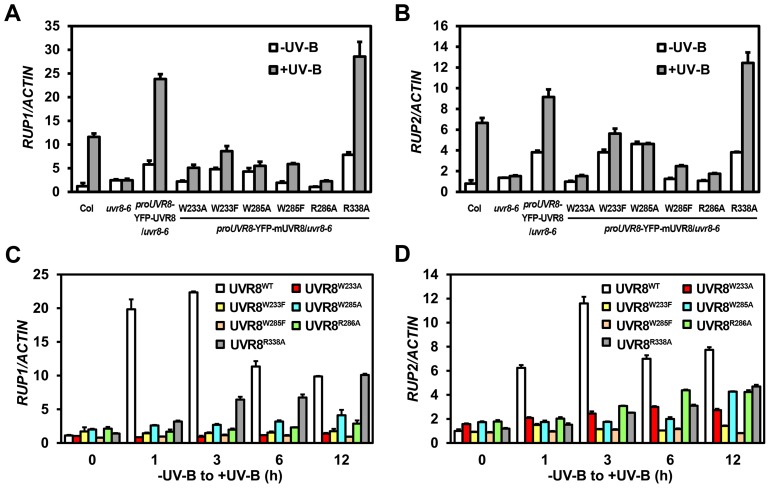
UVR8 variants show defective transcriptional regulation of *RUP1* and *RUP2* by photomorphogenic UV-B. (A–B) qRT-PCR analysis of *RUP1* (A) and *RUP2* (B) in 4-day-old seedlings grown under −UV-B and +UV-B. Data are shown as mean ± SD; n = 3. (C–D) qRT-PCR analysis of *RUP1* (C) and *RUP2* (D) in 4-day-old seedlings transferred from −UV-B to +UV-B and harvested at indicated time points. Data are shown as mean ± SD; n = 3.

Downstream of UVR8-COP1, RUP1 and RUP2 contribute to the negative feedback regulation of UV-B-induced photomorphogenesis. Given that *RUP1* and *RUP2* are known to be induced by photomorphogenic UV-B dependent on UVR8, COP1 and HY5 [19], it was noteworthy that the accumulation of *RUP1* and *RUP2* mRNA was apparently reduced in all UVR8 variants except UVR8^R338A^ (Figures 4A and 4B). As early UV-B responsive genes, *RUP1* and *RUP2* were activated in a temporal manner in response to photomorphogenic UV-B to balance UV-B specific signaling [19]. Using 4-day-old seedlings grown under −UV-B and then transferred to +UV-B for various periods of time, we found that the temporal induction of *RUP1* and *RUP2* by photomorphogenic UV-B was apparently observed in YFP-UVR8^WT^, but was retarded in all the UVR8 variant lines (Figures 4C and 4D). It is worth pointing out that the transcript levels of *RUP1* and *RUP2* were elevated within 1 hours of UV-B irradiation to a peak and then fell back in YFP-UVR8^WT^, whereas they continued to rise to a lower peak particularly in YFP-UVR8^W285A^, YFP-UVR8^R286A^ and YFP-UVR8^R338A^ over the 12-hour UV-B treatment (Figures 4C and 4D). These results suggest that *RUP1* and *RUP2* fail to be activated in our UVR8 variants, and thus do not establish the repressive transcriptional modules required for balanced UV-B signaling. Overall, none of the UVR8 variants are functionally equivalent to YFP-UVR8^WT^, which was consistent with our phenotypic observations.

### UVR8 variants impair UV-B light perception, UVR8 monomerization and UVR8-COP1 association

**Figure 5:**
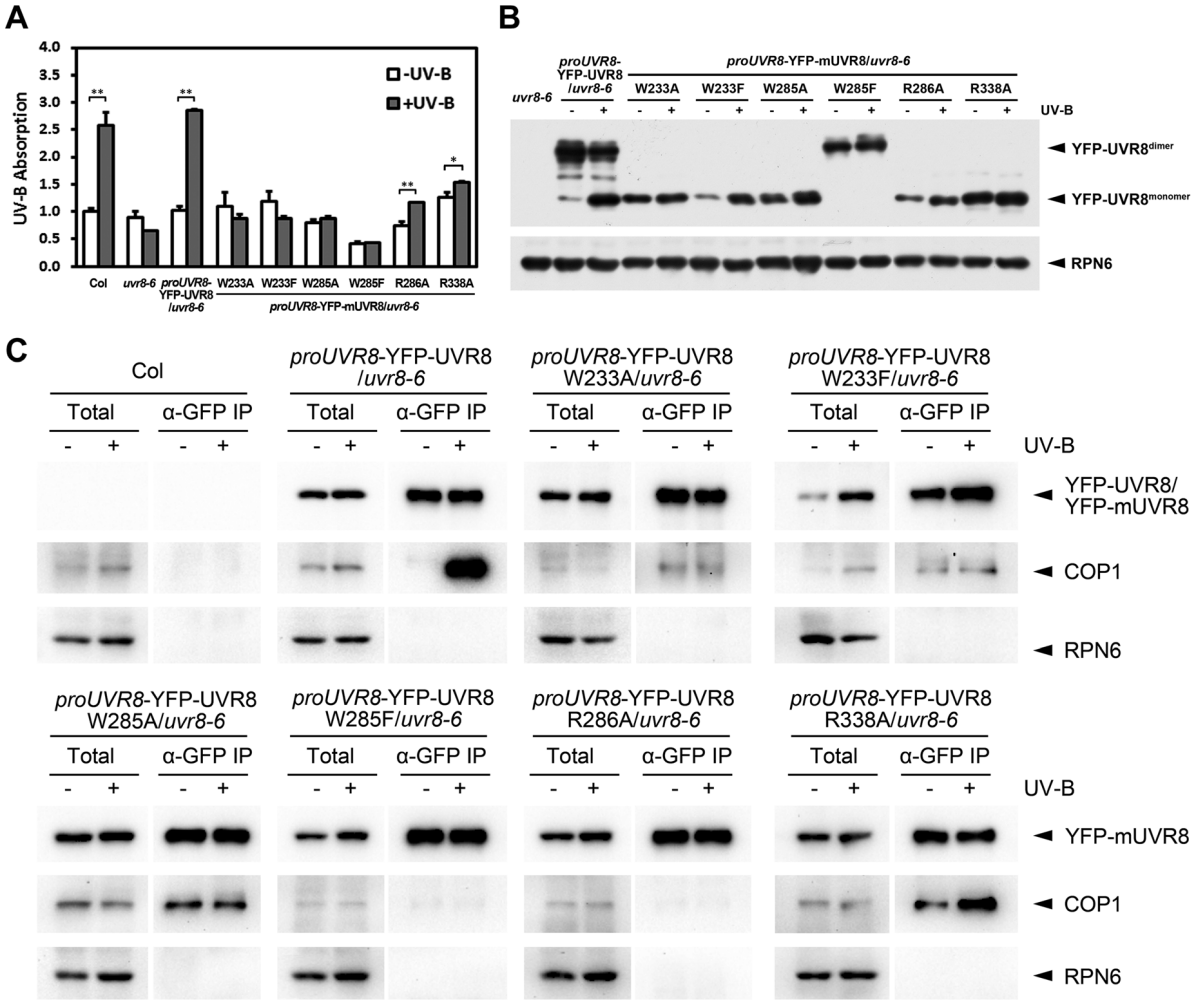
UVR8 mutations affect UV-B light perception, UVR8 monomerization and UVR8-COP1 association. (A) Absorbance at 310 nm of plant total proteins extracted from 4-day-old seedlings grown under −UV-B and +UV-B. Data are shown as mean ± SD; n = 3. *, p<0.05. **, p<0.01. Student's t test. (B) Conformational status of wild-type and mutated UVR8 proteins in 4-day-old seedlings grown under −UV-B and +UV-B. Total plant proteins without heat denaturation were assayed in SDS-PAGE and immunoblot analysis by anti-GFP antibody. Anti-RPN6 was used as a loading control. The asterisks indicate unspecific degradation products. (C) *In vivo* co-immunoprecipitation (co-IP) assays using 4-day-old seedlings grown under −UV-B and +UV-B by anti-GFP antibody. Immunoblot analysis was performed by anti-GFP and anti-COP1 antibodies. Anti-RPN6 was used as a un-immunoprecipitated and loading control.

In order to better understand the biochemical activity of these residues *in vivo*, we investigated the efficiency of UV-B light perception, UVR8 monomerization and the formation of UVR8-containing complex in all of our UVR8 variant plants. By measuring UV-B absorbance at 310 nm, the central wavelength of our photomorphogenic UV-B condition, we found that Col and YFP-UVR8^WT^ exhibited a strong ability to sense UV-B. In contrast, mutations in either residue of the internal chromophore, W233 or W285, led to a complete loss of UV-B absorption. YFP-UVR8^R286A^ and YFP-UVR8^R338A^ retained the ability to sense UV-B, but at significantly diminished levels, which suggests that the disruption of the homodimeric interface has a negative impact on the full activity of UVR8 to perceive UV-B light (Figure 5A).

In SDS-PAGE analysis, we found that in response to photomorphogenic UV-B, while YFP-UVR8^WT^ switched from dimer to monomer, none of YFP-UVR8 variant proteins showed a comparable conformational change. YFP-UVR8^W233A^, YFP-UVR8^W233F^, YFP-UVR8^W285A^, YFP-UVR8^R286A^ and YFP-UVR8^R338A^ were monomeric, while YFP- UVR8^W285F^ was dimeric (Figure 5B). Thus the conformational profiles of wild-type and variant UVR8 observed in *Arabidopsis* were consistent with those found in yeast.

We next examined the endogenous association of UVR8 variants and COP1 by using *in vivo* co-immunoprecipitation (co-IP) assays. In agreement with previous studies [7], [14], YFP-UVR8^WT^ co-immunoprecipitated a high level of COP1 specifically under +UV-B. However, independent of UV-B, YFP-UVR8^W285F^ and YFP-UVR8^R286A^ scarcely co-immunoprecipitated COP1, while YFP-UVR8^W233F^ and YFP-UVR8^W233A^ co-immunoprecipitated very low levels of COP1. YFP-UVR8^W285A^ and YFP-UVR8^R338A^ co-immunoprecipitated medium levels of COP1 under −UV-B, while the latter was also observed to co-immunoprecipitate more COP1 under +UV-B (Figure 5C). These results reveal that the monomeric conformation is not sufficient for UVR8 to associate with COP1 *in vivo*. The relatively close association of YFP-UVR8^W285A^ and YFP-UVR8^R338A^ with COP1 without UV-B treatment indicates that a specific activity of UVR8 might be produced by W285A or R338A mutation.

### Constitutive activity of UVR8 is dependent on constant UVR8-COP1 interaction

**Figure 6:**
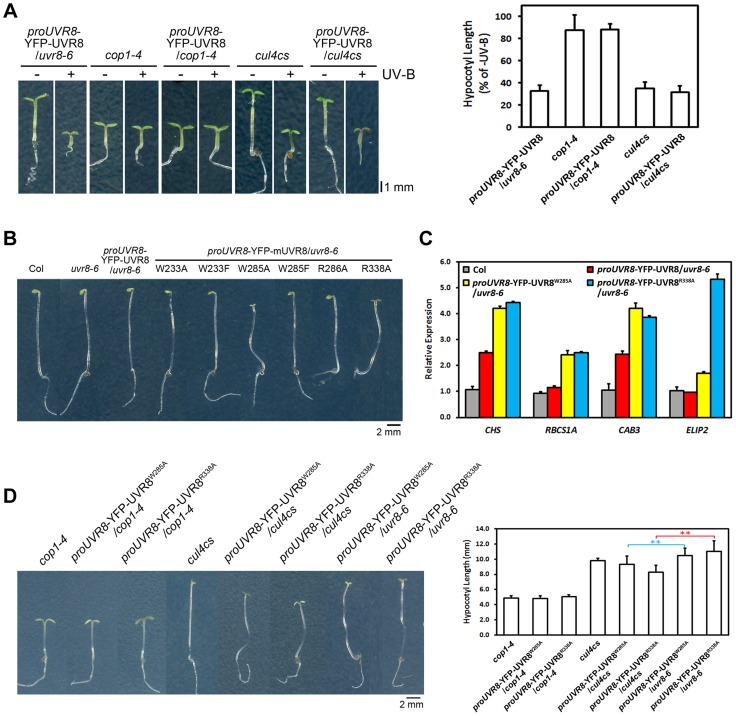
UVR8^W285A^ and UVR8R^338A^ display constitutive activity in darkness. (A) Phenotypes and relative hypocotyl length of 4-day-old seedlings of indicated genotypes grown under −UV-B and +UV-B. Data are shown as mean ± SD; n>30. (B) Phenotypes of 4-day-old dark-grown seedlings. Data are shown as mean ± SD; n>30. (C) qRT-PCR analysis of light-regulated gene expression in 4-day-old dark-grown seedlings of indicated genotypes. Data are shown as mean ± SD; n = 3. (D) Phenotypes and hypocotyl length of 4-day-old dark-grown seedlings of indicated genotypes. Data are shown as mean ± SD; n>30. **, p<0.01. Student's t test.

As both *cop1-4* and *uvr8-6* suffer from diminished gene expression, hypocotyl growth, anthocyanin accumulation and acclimation in response to photomorphogenic UV-B, COP1 and UVR8 share a high degree of functional similarity in photomorphogenic UV-B signaling [14], [25]. We found that YFP-UVR8^WT^/*cop1-4* phenocopied *cop1-4* (Figure 6A), clearly demonstrating that COP1 acts genetically downstream of UVR8. The observation that YFP-UVR8^WT^ failed to rescue *cop1-4* (Figure 6A) also suggests that the function of UVR8 is dependent on COP1. Though CUL4 works in concert with COP1 in darkness, it functionally disassociates from COP1 and plays a negative role in UV-B-induced photomorphogenesis [17]. Since *cul4cs* exhibited no obvious defect in hypocotyl growth under UV-B [17], we consistently found that YFP-UVR8^WT^/*cul4cs* phenocopied YFP-UVR8^WT^ and *cul4cs* (Figure 6A).

We have previously proposed that the UVR8-COP1 interaction mediated by UV-B enables a reorganization of COP1 complexes and eventually results in a functional switch of COP1 from a repressor to a promoter of photomorphogenesis [17]. Both the enhanced photomorphogenesis of YFP-UVR8^W285A^ and YFP-UVR8^R338A^ under −UV-B (Figures 2A, 2B and 2C) and the persistent binding of UVR8^W285A^ and UVR8^R338A^ (Figures 1B and 5C) to COP1 prompted us to examine the development of all the UVR8 variants in darkness. In addition to Col, *uvr8-6* and YFP-UVR8^WT^, YFP-UVR8^W233A^, YFP-UVR8^W233F^, YFP-UVR8^W285F^ and YFP-UVR8^R286A^ all showed typical skotomorphogenic responses, demonstrating long hypocotyls, closed cotyledons and apical hooks. In contrast, YFP-UVR8^W285A^ and YFP-UVR8^R338A^ displayed open cotyledons, suggesting that these two UVR8 variants were capable of inducing constitutive photomorphogenesis irrespective of their exposure to light (Figure 6B). We then examined light-regulated gene expression, and found highly accumulated transcripts of *CHS*, *RIBULOSE BISPHOSPHATE CARBOXYLASE SMALL CHAIN 1A* (*RBCS1A*), *CHLOROPHYLL A/B BINDING PROTEIN 3* (*CAB3*) and *ELIP2*, in YFP-UVR8^W285A^ and YFP-UVR8^R338A^ (Figure 6C). Though UVR8^W233A^, UVR8^W233F^ and UVR8^R286A^ had constitutive physical interactions with COP1 in yeast (Figure 1B), their affinity with COP1 *in vivo* was much weaker than that of UVR8^W285A^ and UVR8^R338A^ (Figure 5C), indicating that conversion of COP1's function might require a threshold level of UVR8-COP1 interaction.

We have also pointed out that in response to UV-B, monomerized UVR8 might sequester COP1 from the CUL4-DDB1 based E3 apparatus [17]. In darkness, compared with YFP-UVR8^W285A^/*uvr8-6* and YFP-UVR8^R338A^/*uvr8-6*, YFP-UVR8^W285A^/*cop1-4* and YFP-UVR8^R338A^/*cop1-4* mimicked *cop1-4* (Figure 6D), which is consistent with our conclusion that UVR8 functions in a COP1-dependent manner. YFP-UVR8^W285A^/*cul4cs* and YFP-UVR8^R338A^/*cul4cs* exhibited enhanced constitutive photomorphogenesis with decreased hypocotyl length (Figure 6D). These results indicate that the reduced CUL4 protein abundance might facilitate the release of an increased amount of COP1 from CUL4-DDB1 to allow an improved association between UVR8 and COP1, and in turn achieve a highly switched function of COP1 in promoting photomorphogenesis.

## Discussion

### Light-absorbing tryptophans and dimer-stabilizing arginines are intrinsically coordinated to fulfill UVR8 function

The molecular framework of UV-B-induced photomorphogenesis has been gradually established over the past ten years. For example, both the identification of UVR8 as a UV-B photoreceptor and the subsequent structural analysis of recombinant UVR8 have recently led to a proposed mechanism of UVR8-dependent UV-B signaling initiation [7], [15], [16]. A series of UVR8 variant proteins have been generated to further demonstrate that UVR8 exploits its own light-absorbing tryptophans and dimer-stabilizing arginines to perceive light and initiate protein conformational changes, respectively [15], [16]. UVR8^W233F^, UVR8^W285A^ and UVR8^W285F^ are lack of the ability to perceive UV-B *in vitro* [16]. UVR8^W285A^ is at least partially monomeric and interacts with COP1. UVR8^W285F^, on the other hand, is dimeric and unable to interact with COP1 in yeast, plant and mammalian cells [7], [15], [16], [22], [26]. UVR8^R286A^ and UVR8^R338A^ are monomeric and display an obviously diminished perception of UV-B light *in vitro* [16]. In *Arabidopsis*, mutations in UVR8's tryptophan residues have been pointed out to result in the physical impairment of photomorphogenic UV-B responses [22]. However, the exact mechanism driving this hindered response remains unknown. Moreover, it is far less understood concerning the biological significance of light-absorbing and dimer-stabilizing residues in UVR8, as well as the signaling process that connects UV-B light perception, UVR8 monomerization and subsequent signaling events including the organization of UVR8-COP1-SPA complex(es).

In our study, we generated transgenic plants expressing UVR8 variant proteins under *UVR8*'s native promoter, based on the site-directed mutagenesis of two light-absorbing tryptophans and two dimer-stabilizing arginines. These variant proteins drove varied levels of UV-B-induced photomorphogenesis. All the variants were observed to impair both UVR8's activity to perceive UV-B light and its ability to undergo a dimer-to-monomer transition (Figures 5A and 5B), suggesting the reciprocal impacts by these residues involved in the same intramolecular interaction network. More severe phenotypic defects were observed in UV-B-absorbing variants than those in UVR8-dimerizing variants (Figures 2A, 2B and 2C), confirming that UV-B light perception precedes UVR8 monomerization to launch UV-B signaling. In addition, the unchangeable conformational status of the UVR8 variants (Figure 5B) abolished the dimer-monomer-dimer cycling of UVR8, and further disturbed the balance in UV-B signaling. Molecularly, all the mutations caused an altered hierarchy of UV-B responsive gene expression (Figures 3 and 4). They failed to accurately establish the promotive module formed by the UVR8-COP1-HY5 core pathway and the negative transcriptional feedback mediated by RUP1 and RUP2, leading to inadequate and unbalanced photomorphogenic UV-B responses. Taken together, the roles of light-absorbing tryptophans and dimer-stabilizing arginines in UVR8 are intrinsically coordinated for UVR8 activity in UV-B-induced photomorphogenesis.

### A threshold UVR8-COP1 interaction plays a critical role in coupling with UV-B light perception to signal transduction

The direct interaction between UVR8 and COP1 takes place rapidly following UV-B light perception and UVR8 monomerization [7]. It requires UVR8 to be in its monomeric form [7] whereas physically monomeric UVR8 is not sufficient for the formation of the UV-B-dependent COP1 complex and to mediate photomorphogenesis in response to UV-B in plants (Figures 2A and 5C). Though this point has been previously articulated [22], the molecular mechanism underlying this empirical observation is still unknown. This discrepancy serves to indicate that factors downstream of UV-B light perception and UVR8 monomerization might essentially govern the progression of UV-B signaling.

In terms of the transcriptome reprogramming induced by photomorphogenic UV-B, *cop1-4* resulted in the loss of transcriptional responses of a broader range of genes than *uvr8-6* [14]. While UVR8's ability to mediate UV-B-induced photomorphogenesis was observed to be dependent on COP1 (Figure 6A), COP1 did not appear to significantly influence UVR8 conformation [7], [20]. These data collectively indicate that COP1 is at least as essential as UVR8 in photomorphogenic UV-B signaling, if not superior to UVR8, due to its role in the formation of UVR8-COP1-SPA complex(es). By using the native promoter of *UVR8* to drive UVR8 variants in plants, we selected those transgenic lines expressing comparable levels of UVR8 variant proteins (Figure 2D), in order to stringently analyze each variant in parallel, particularly to examine the *in vivo* association intensity between UVR8 and COP1 (Figure 5C). As a result, we propose that a threshold level of the *in vivo* association between UVR8 and COP1 is critically required for photomorphogenic UV-B signaling output, founded on the following evidence. Firstly, UVR8 monomers, rather than UVR8-COP1 interaction, were detected in −UV-B-treated plant and yeast cells expressing wild-type UVR8 (Figures 1, 5B and 5C). Secondly, the *in vivo* levels of UVR8-COP1 association correspond with the physiological and molecular features of the transgenic UVR8 variant lines (Figures 2A and 5C). Only those UVR8 variants that possess high affinity with COP1 in plants, namely YFP-UVR8^WT^ under +UV-B, and YFP-UVR8^W285A^ and YFP-UVR8^R338A^ under −UV-B and +UV-B in our study, result in photobiological activity in specific light contexts (Figures 5C, 2A and 6B). Thirdly, once the affinity between UVR8 and COP1 is conditionally increased, such as the situation in *cul4cs* that might release more COP1 for UVR8 to interact with, the photobiological activity of UVR8 is ultimately enhanced (Figure 6D). In agreement with our hypothesis, a most recent report has presented that comparing with the overexpressed wild-type UVR8 (UVR8-OX), UVR8^W285A^ leads to improved photomorphogenesis and UV-B tolerance by increased COP1 binding affinity, though UVR8^W285A^ and UVR8-OX express equivalent UVR8 proteins [27].

Though our UVR8 variants do not demonstrate equivalent patterns of interaction with COP1 in yeast and plants (Figures 1B and 5C), this discrepancy can most likely be explained by the fact that the yeast two-hybrid system is devoid of any other factors that might influence the UVR8-COP1 interaction. The variation in the UVR8 variants' affinity for COP1 observed in plants may be due to a wide variety of factors. For example, UVR8 monomer variants might undergo protein folding processes in plants that are distinct from those in yeast, given that each mutated residue locates specifically in the intramolecular interaction network (Figure S1A). Similarly, the protein levels of endogenous COP1 vary amongst the different UVR8 monomer variants (Figure 3G). Finally, a number of the other proteins present *in vivo* might interact with COP1 and/or UVR8 in manner that either enhances or hinders the *in vivo* contact of UVR8 and COP1. Further research is required, however, in order to fully disentangle and elucidate the impact of the great complexity *in vivo*.

### Constitutive light signaling is mediated by UVR8^W285A^ and UVR8^R338A^

Two constitutively active forms of UVR8, UVR8^W285A^ and UVR8^R338A^ have been uncovered in our study. Though they differed in the phenotypic features of UV-B-induced photomorphogenesis, they both displayed constitutive interaction with COP1 (Figure 5C) and photomorphogenic development in darkness (Figure 6B). It is worth pointing out that a previous report did not find constitutive photomorphogenesis in GFP-UVR8^W285A^ [22] whereas a recent independent study reached a consensus with ours by showing the constitutive photomorphogenesis in UVR8^W285A^ [27].

Structure of dimeric UVR8 has revealed that W285, the UV-B chromophore, is located at the center of the strong cation-π and π-π interaction network at the dimer interface, while R338, responsible for dimer stabilization, is located at the edge of the interaction network [16]. It is suggested that W285 is more essential to the function of UVR8 than R338. Hence it is reasonable that YFP-UVR8^W285A^ failed to respond to photomorphogenic UV-B while YFP-UVR8^R338A^ was still able to, in terms of UV-B light perception, UV-B-induced UVR8-COP1 association and gene expression, and eventually photomorphogenic development (Figures 2A–C, 3A–C, 5A and 5C). On the other hand, in the absence of UV-B, YFP-UVR8^W285A^ and YFP-UVR8^R338A^ associated with a sufficient amount of COP1, and thus promoted hypocotyl growth, anthocyanin accumulation and gene activation under −UV-B (Figures 2A–C, 3A–C and 5C), and even photomorphogenesis in darkness (Figures 6B and 6C).

As reported, other gain-of-function alleles of photoreceptors were also produced via point mutations in their photosensory regions, such as the GAF domain tyrosine mutants of phytochromes (PHY), PHYA^Y242H^ and PHYB^Y276H^ [28], and the photolyase-related (PHR) domain glycine mutants of cryptochromes (CRY), CRY1^G380R^, CRY2^G377R^ [29]. PHYB^Y276H^ was proposed to interact with COP1 in a light-independent manner to diminish the degradation of HY5 by COP1 [28]. CRY1^G380R^ was found to co-localize in nucleus with COP1 to promote the nuclear exclusion of COP1 in darkness [29]. In contrast to its role in the repression of photomorphogenesis induced by far-red and visible light [30], COP1 is known to promote UV-B-induced photomorphogenesis [25]. Upon UV-B irradiation, COP1 rapidly interacts with UVR8 in nucleus [7], and switches from degrading to stabilizing HY5 [17], [31]. The constitutive photomorphogenesis in YFP-UVR8^W285A^ and YFP-UVR8^R338A^ is not resulted from a loss in COP1 protein abundance that was observed in *cop1-4* (Figure S3). Therefore, the way UVR8 modulates COP1 dramatically differs from the activity repression of COP1 by cryptochromes or phytochromes. Furthermore, UVR8 is involved in diverse developmental processes in plants. Photomorphogenic UV-B signaling displays crosstalk with circadian regulation [32], and it also controls leaf morphogenesis [33], [34], drought tolerance [35] and plant immune response [36]. Beyond the area of plant researches, UVR8 has also been implemented in optical control of protein interactions [26], [37] and multi-chromatic expression regulation [38] in mammalian cells. Thus, the characterization of constitutively active UVR8 variants should serve to elucidate the mechanism of UV-B specific signaling in plants and advance protein engineering pertinent to a variety of medical applications.

## Materials and Methods

### Plant materials and growth conditions

The wild-type *Arabidopsis thaliana* used in this study is of the Columbia (Col) ecotype. Some of the mutants and transgenic lines used in this study were described previously: *cop1-4* [39], *uvr8-6* [14], *cul4cs* [40], *proUVR8*-YFP-UVR8/*uvr8-6*, *proUVR8*-YFP-UVR8^W285A^/*uvr8-6* and *proUVR8*-YFP-UVR8^W285F^/*uvr8-6* [17].

The vectors for UVR8 variant transgenic lines, *proUVR8*-YFP-mUVR8/*uvr8-6*, were generated using the QuikChange site-directed mutagenesis kit (Stratagene). The primers are listed in Table S1. These transgenic lines were prepared using floral dipping method [41].

The *Arabidopsis* materials were grown as described previously [24]. The seeds were surface-sterilized and sown on solid Murashige and Skoog medium supplemented with 1% sucrose for biochemical assays or with 0.3% sucrose for phenotypic analysis, and cold treated at 4°C for 4 days. For photomorphogenic UV-B treatment, seedlings were grown at 22°C under continuous white light (3 µmol·m^−2^·s^−1^, measured by LI-250 Light Meter, LI-COR Biosciences) supplemented with Philips TL20W/01RS narrowband UV-B tubes (1.5 µmol·m^−2^·s^−1^, measured by TN-340 UV-B Light Meter, China) under a 350-nm cutoff (half-maximal transmission at 350 nm) filter ZUL0350 (−UV-B; Asahi spectra, USA) or a 300-nm cutoff (half-maximal transmission at 300 nm) filter ZUL0300 (+UV-B; Asahi spectra, USA).

### UVR8 dimer/monomer assay

For the assay in yeast, the vectors of LexA fused wild-type and mutated UVR8 were transformed into the yeast strain EGY48 (Clontech). Total proteins were extracted from transformants in Yeast Protein Extraction Reagent (Thermo), 1 mM phenylmethylsulfonyl fluoride (PMSF), and 1× complete protease inhibitor cocktail (Roche), and then kept on ice under −UV-B (3 µmol·m^−2^·s^−1^ of white light) or +UV-B (3 µmol·m^−2^·s^−1^ of white light and 1.5 µmol·m^−2^·s^−1^ of UV-B) for 20 min. Added with 4× loading buffer containing 250 mM Tris-HCl (pH 6.8), 2% SDS, 20% β-mercaptoethanol, 40% glycerol, and 0.5% bromophenol blue, the samples were subjected to immunoblot analysis without boiling.

The assay in plants was performed as previously described [17]. Total proteins were extracted from 4-day-old *Arabidopsis* seedlings grown under −UV-B or +UV-B in protein extraction buffer containing 50 mM Tris-HCl (pH 7.5), 150 mM NaCl, 1 mM EDTA, 10% glycerol, 0.1% Tween 20, 1 mM phenylmethylsulfonyl fluoride (PMSF), and 1× complete protease inhibitor cocktail (Roche). Then the cell extracts were kept on ice under exactly the same condition (−UV-B or +UV-B) as where the seedlings were grown for 30 min. Added with 4× loading buffer containing 250 mM Tris-HCl (pH 6.8), 2% SDS, 20% β-mercaptoethanol, 40% glycerol, and 0.5% bromophenol blue, the samples were subjected to immunoblot analysis without boiling.

### Yeast two-hybrid assay

The respective combinations of vectors were cotransformed into the yeast strain EGY48 (Clontech) containing the reporter plasmid *p8op::LacZ*. Transformants were grown under −UV-B (3 µmol·m^−2^·s^−1^ of white light) and +UV-B (3 µmol·m^−2^·s^−1^ of white light and 1.5 µmol·m^−2^·s^−1^ of UV-B) on proper dropout plates containing X-gal (5-bromo-4-chloro-3-indolyl-β-D-galactopyranoside) for blue color development.

### Hypocotyl and anthocyanin measurement

Hypocotyl length was measured as previously described [24]. For each line grown under −UV-B or +UV-B for 4 days, hypocotyl length was analyzed in three biological replicates. In each replicate, at least 30 *Arabidopsis* seedlings were measured. The relative hypocotyl length was presented as the percentage of the hypocotyl length under +UV-B with respect to that under −UV-B (% of −UV-B). For each line grown in darkness, hypocotyl length was analyzed using at least 30 *Arabidopsis* seedlings. The quantification of hypocotyl length was performed by ImageJ (http://rsb.info.nih.gov/ij/).

Anthocyanin was extracted and quantified as previously described [42]. Briefly, *Arabidopsis* seedlings were harvested and placed into extraction solution (18% 1-propanol and 1% HCl), and boiled for 3 minutes. Then the mixture was left in darkness for at least 3 hours at room temperature. After a brief centrifugation to pellet the tissue debris, the supernatant was removed and diluted with the extraction solution. The anthocyanin content was presented as A_535_−2(A_650_) g^−1^ fresh weight.

### Quantitative real-time PCR

Total RNA was extracted from 4-day-old *Arabidopsis* seedlings grown under −UV-B or +UV-B using the RNeasy plant mini kit (Qiagen). Reverse transcription was performed using SuperScript II first-strand cDNA synthesis system (Invitrogen) according to the manufacturer's instructions. Real-time qPCR analysis was performed using SYBR Premix Ex Taq (Takara) with Applied Biosystems 7500 Real-Time PCR System. Each experiment was repeated with three independent samples, and RT-PCR reactions were performed in three technical replicates for each sample. The primers are listed in Table S1.

### Measurement of UV-B absorbance

Total proteins was extracted from 4-day-old *Arabidopsis* seedlings grown under −UV-B and +UV-B in protein extraction buffer containing 50 mM Tris-HCl (pH 7.5), 150 mM NaCl, 1 mM PMSF, and 1× complete protease inhibitor cocktail (Roche). Absorbance at 310 nm of plant total proteins adjusted to equal concentration and total amount were measured. Each experiment was repeated with three independent samples.

### Co-immunoprecipitation assays and immunoblot analysis

1 mg of total proteins was extracted from 4-day-old *Arabidopsis* seedlings in protein extraction buffer containing 50 mM Tris-HCl (pH 7.5), 150 mM NaCl, 1 mM EDTA, 10% glycerol, 0.1% Tween 20, 1 mM PMSF, and 1× complete protease inhibitor cocktail (Roche). The extracts were incubated with 8 µl anti-GFP antibodies (Invitrogen) coupled with 25 µl Dynabeads Protein G (Invitrogen) for 3 hours at 4°C under the same condition (−UV-B or +UV-B) as where the seedlings were grown. Then the dynabeads were washed three times by protein extraction buffer. Next the precipitates were eluted into 100 mM Glycine (pH 2.5) and 100 mM NaCl, and immediately neutralized by 2 M Tris-HCl (pH 9.0) and 100 mM NaCl, and finally concentrated using Strataresin (Stratagene) before immunoblot analysis. Primary antibodies used in this study were anti-COP1 and anti-RPN6 [40], anti-HY5 [31], anti-GFP (Invitrogen) and anti-UVR8 [17] antibodies.

## Supporting Information

Figure S1 UVR8 variants display altered interaction with RUP1 and RUP2 in yeast. (A) A schematic diagram of UVR8 mutations generated in this study. Residues targeted for point mutations are in color. Blue and red represent positive and negative charges respectively. Purple circles show cation-π or π-π interactions. The size of purple cycles indicates the intensity of interactions. Arrows denote point mutations generated in this study. (B) Interaction of wild-type and mutated UVR8 proteins with RUP1 and RUP2 in yeast two-hybrid assays. Transformants in the respective combinations were incubated under −UV-B and +UV-B for 16 h.
(TIF)

Figure S2 Protein levels and UV-B-induced hypocotyl growth in transgenic lines of UVR8 variants. (A) Immunoblot assay of endogenous and transgenic UVR8 proteins (by anti-UVR8 antibody) in 4-day-old seedlings grown under −UV-B and +UV-B. Anti-RPN6 was used as a loading control. The asterisk indicates an unspecific cross-reactive band. (B) Hypocotyl length of 4-day-old seedlings of transgenic UVR8 variant lines grown under −UV-B and +UV-B. Two independent transgenic lines for each UVR8 variant were analyzed. Data are shown as mean ± SD; n>30. (C) Immunoblot assay of wild-type and mutated UVR8 proteins (by anti-GFP antibody) in 4-day-old transgenic seedlings grown under −UV-B and +UV-B. Two independent transgenic lines for each UVR8 variant were analyzed. Anti-RPN6 was used as a loading control.
(TIF)

Figure S3 COP1 proteins are abundant in constitutively active UVR8 variant lines. Immunoblot assay of COP1 proteins (by anti-COP1 antibody) in 4-day-old seedlings grown in darkness. Anti-RPN6 was used as a loading control.
(TIF)

Table S1 Summary of primers used in this study.
(DOCX)
