# Supplementary figures and images for: Photoactivated UVR8-COP1 Module Determines Photomorphogenic UV-B Signaling Output in Arabidopsis
Source: PLoS Genet. 2014 Mar 20;10(3):e1004218. doi: 10.1371/journal.pgen.1004218 (PMC3961177; doi:10.1371/journal.pgen.1004218)

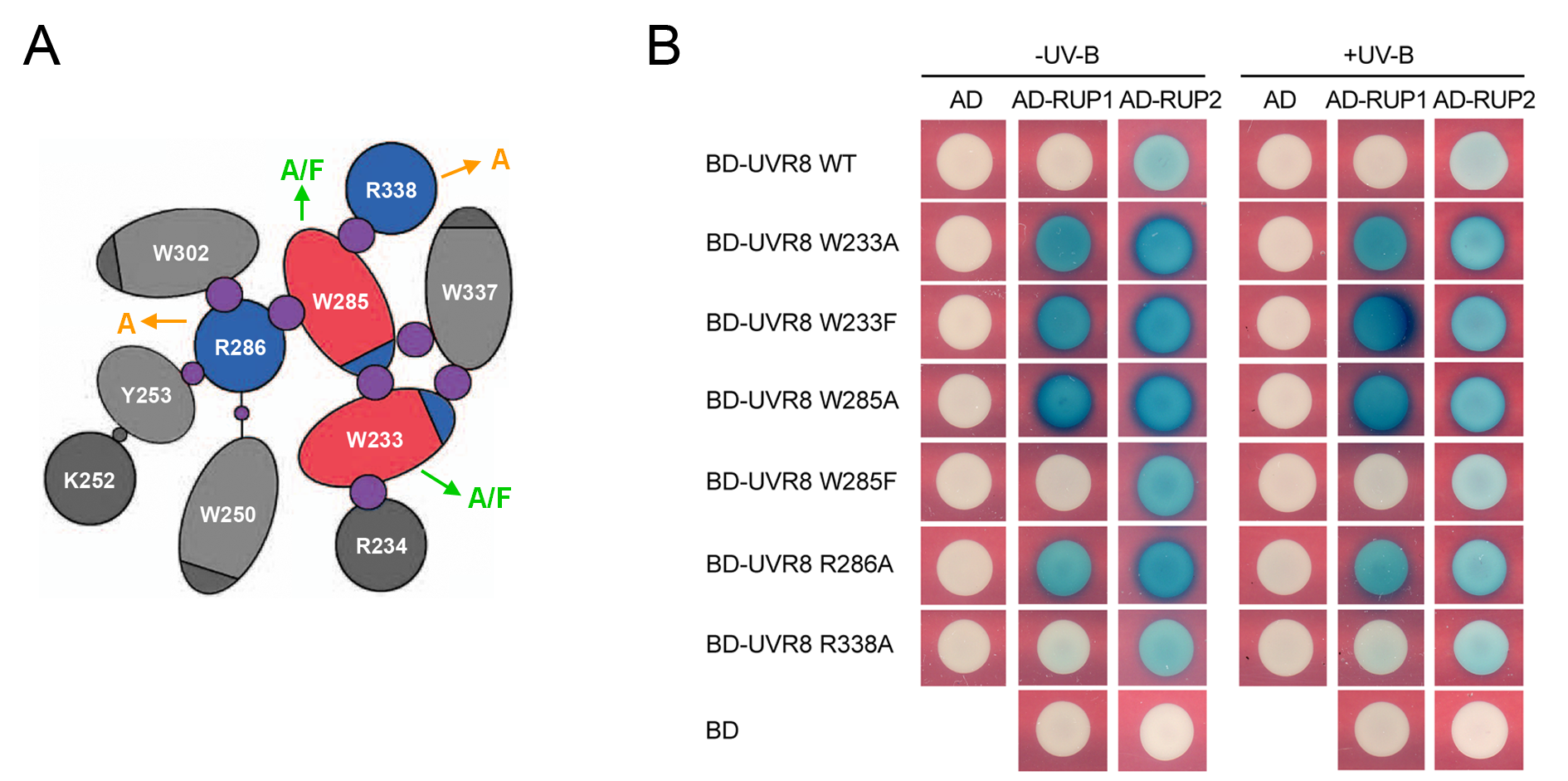

Supplement: Figure S1 — UVR8 variants display altered interaction with RUP1 and RUP2 in yeast. (A) A schematic diagram of UVR8 mutations generated in this study. Residues targeted for point mutations are in color. Blue and red represent positive and negative charges respectively. Purple circles show cation-π or π-π interactions. The size of purple cycles indicates the intensity of interactions. Arrows denote point mutations generated in this study. (B) Interaction of wild-type and mutated UVR8 proteins with RUP1 and RUP2 in yeast two-hybrid assays. Transformants in the respective combinations were incubated under −UV-B and +UV-B for 16 h. (TIF) [file pgen.1004218.s001.tif]

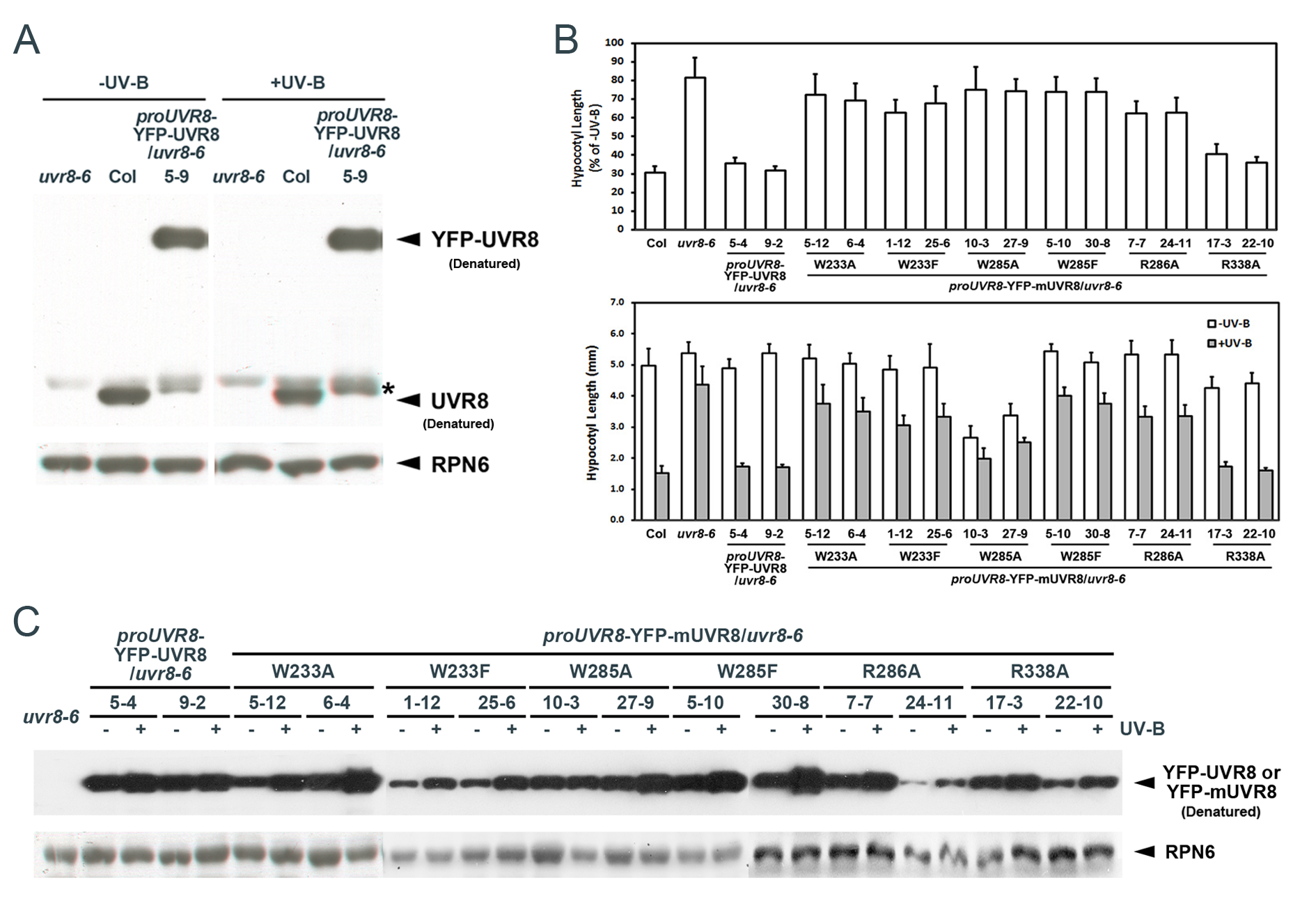

Supplement: Figure S2 — Protein levels and UV-B-induced hypocotyl growth in transgenic lines of UVR8 variants. (A) Immunoblot assay of endogenous and transgenic UVR8 proteins (by anti-UVR8 antibody) in 4-day-old seedlings grown under −UV-B and +UV-B. Anti-RPN6 was used as a loading control. The asterisk indicates an unspecific cross-reactive band. (B) Hypocotyl length of 4-day-old seedlings of transgenic UVR8 variant lines grown under −UV-B and +UV-B. Two independent transgenic lines for each UVR8 variant were analyzed. Data are shown as mean ± SD; n>30. (C) Immunoblot assay of wild-type and mutated UVR8 proteins (by anti-GFP antibody) in 4-day-old transgenic seedlings grown under −UV-B and +UV-B. Two independent transgenic lines for each UVR8 variant were analyzed. Anti-RPN6 was used as a loading control. (TIF) [file pgen.1004218.s002.tif]

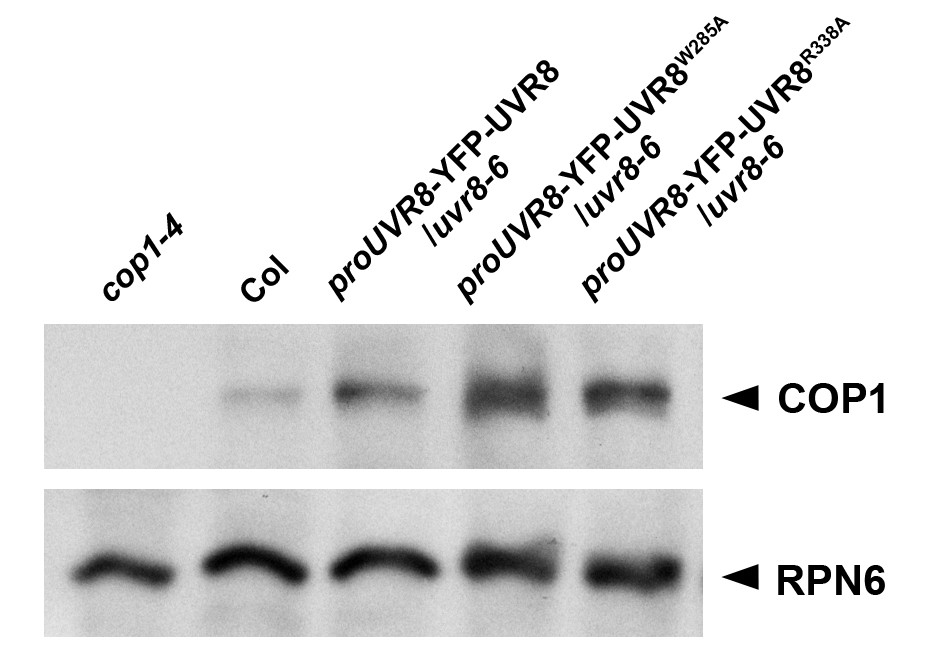

Supplement: Figure S3 — COP1 proteins are abundant in constitutively active UVR8 variant lines. Immunoblot assay of COP1 proteins (by anti-COP1 antibody) in 4-day-old seedlings grown in darkness. Anti-RPN6 was used as a loading control. (TIF) [file pgen.1004218.s003.tif]
