# Supplementary material for: Photoactivated UVR8-COP1 Module Determines Photomorphogenic UV-B Signaling Output in Arabidopsis
Source: PLoS Genet. 2014 Mar 20;10(3):e1004218. doi: 10.1371/journal.pgen.1004218 (PMC3961177; doi:10.1371/journal.pgen.1004218)
Supplement: Table S1 — Summary of primers used in this study. (DOCX) [file pgen.1004218.s004.docx]

**Table S1** Summary of primers used in this study.

| Site-directed Mutagenesis of UVR8 | |
| --- | --- |
| UVR8 W233A FP | TGTCAATGGTTGCTTGTGGAGCGCGGCACACAATATCAGTTTCCT |
| UVR8 W233A RP | AGGAAACTGATATTGTGTGCCGCGCTCCACAAGCAACCATTGACA |
| UVR8 W233F FP | TGTCAATGGTTGCTTGTGGATTTCGGCACACAATATCAGTTTCCT |
| UVR8 W233F RP | AGGAAACTGATATTGTGTGCCGAAATCCACAAGCAACCATTGACA |
| UVR8 W285A FP | TCTCCCAGATTTCGGGAGGTGCGAGACATACAATGGCATTGACTT |
| UVR8 W285A RP | AAGTCAATGCCATTGTATGTCTCGCACCTCCCGAAATCTGGGAGA |
| UVR8 W285F FP | TCTCCCAGATTTCGGGAGGTTTTAGACATACAATGGCATTGACTT |
| UVR8 W285F RP | AAGTCAATGCCATTGTATGTCTAAAACCTCCCGAAATCTGGGAGA |
| UVR8 R286A FP | TCTCCCAGATTTCGGGAGGTTGGGCACATACAATGGCATTGACTT |
| UVR8 R286A RP | AAGTCAATGCCATTGTATGTGCCCAACCTCCCGAAATCTGGGAGA |
| UVR8 R338A FP | TAGTTCAAGTCTCATGTGGATGGGCACATACCTTGGCTGTCACTG |
| UVR8 R338A RP | CAGTGACAGCCAAGGTATGTGCCCATCCACATGAGACTTGAACTA |
| Yeast Two-hybrid Assay | |
| UVR8 5' EcoRI | ATACGAATTCATGGCGGAGGATATGGCT |
| UVR8 3' XhoI SacI | ATACCTCGAGGAGCTCTCAAATTCGTACACGCTT |
| RUP1 5' MfeI | ATACCAATTGATGGAGGCTTTGTTCTGCTC |
| RUP1 3' XhoI | ATACCTCGAGTTAGCTTTGTTTGCCCGAGA |
| RUP2 5' MfeI | ATACCAATTGATGAACACTCTTCATCCTCA |
| RUP2 3' XhoI | ATACCTCGAGCTATGGTTTTCTTTTGCCCA |
| qRT-PCR | |
| ACTIN FP | CAAGGCCGAGTATGATGAGG |
| ACTIN RP | GAAACGCAGACGTAAGTAAAAAC |
| ELIP2 FP | CACCACAAATGCCACAGTCT |
| ELIP2 RP | TGCTAGTCTCCCGTTGATCC |
| UGT84A1 FP | AGTCGGGTTTATCGTTCT |
| UGT84A1 RP | ATCCCTTTACCTTTAGCAC |
| CHS FP | ACGTCACGTGTTGAGCGAGTATGG |
| CHS RP | GAGGAACGCTGTGCAAGACGACTG |
| COP1 FP | AGTGTAGTACGGAGGGAAGG |
| COP1 RP | CAATGTTGGCTGAATGAAAT |
| HY5 FP | TCAGAACGAGAACCAGATGCTTAG |
| HY5 RP | TTAGAACCACCACCACCTCCTC |
| RUP1 FP | CGTGGAGAAACAAGTGC |
| RUP1 RP | TCGTCGGAGCCTGA |
| RUP2 FP | TCGGATGACGGGACT |
| RUP2 RP | GACGCAACAAACAGCA |
| RBCS1A FP | CGCAAGGCTAACAACG |
| RBCS1A RP | TCGGAATCGGTAAGGTC |
| CAB3 FP | CGAGGACTTGCTTTACCC |
| CAB3 RP | TGACGATGGCTTGAACG |

FP, forward primer. RP, reverse primer.
